# Altered gut microbiota in erectile dysfunction patients: a pilot study

**DOI:** 10.3389/fmicb.2025.1530014

**Published:** 2025-06-05

**Authors:** Quanxin Su, Kenan Wang, Yayin Luo, Qizhen Tang

**Affiliations:** ^1^Department of Urology, The First Affiliated Hospital of Dalian Medical University, Dalian, Liaoning, China; ^2^Department of Neurology, The First Affiliated Hospital of Dalian Medical University, Dalian, Liaoning, China

**Keywords:** Bacteroides intestinalis, erectile dysfunction, gut microbiota, metabolic pathways, macrogenomics

## Abstract

**Purpose:**

With the growing body of research on gut microbiota in recent years, various potential associations between gut microbiota and health or disease have been identified. However, the role of gut microbiota in Erectile dysfunction (ED) remains poorly understood. This study aimed to compare the changes in gut microbiota and metabolic pathways between ED males and healthy control group, contributing to the exploration of ED pathogenesis.

**Methods:**

Fecal samples were collected from 19 ED patients and 15 healthy controls (aged from 18 to 60 years), with erectile function assessed using the 5-item version of the International Index of Erectile Function (IIEF-5). Macro-genomic sequencing was performed on the NovaSeq PE 150 platform to characterize the gut microbiota distribution among the groups.

**Results:**

No significant differences in alpha diversity of the gut microbiota were observed between the ED and control groups. Additionally, Principal component analysis (PCA) analysis revealed no notable changes in microbiota composition between the two groups. A comparison of the abundance of key species showed that, in the ED group, species such as Ruminococcus gnavus, Thomasclavelia ramosa, Clostridium sp. AF32-12BH, Clostridium nexile, and Eubacterium siraeum were more abundant, while the abundance of Bacteroides intestinalis was decreased compared to the control group. Furthermore, pathways related to nucleotide and lipid metabolism were found to be highly expressed in the ED group.

**Conclusion:**

This pilot study found a decrease in the abundance of Bacteroides intestinalis and an increase in the abundance of Ruminococcus gnavus in the ED sample. These microbiota changes may contribute to ED by promoting atherosclerosis and inhibiting the degradation of branched-chain amino acids. In the future, it may be possible to achieve better outcomes for ED patients by precisely regulating the gut microbiota.

## 1 Introduction

Erectile dysfunction (ED) is a prevalent condition among the male population, with prevalence rates ranging from 37.2 to 48.6% (Goldstein et al., 2020). It is characterized by the persistent inability to achieve or maintain an erection sufficient for a fulfilling sexual life, leading to significant negative impacts on a patient’s psychological well-being and marital relationships (Muneer et al., 2014). In response to the growing emphasis on quality of life, the therapeutic approach to ED has evolved beyond merely improving erectile function to include early diagnosis and the identification of risk factors.

Gut microbiota, a dynamic and diverse community, plays a crucial role in maintaining both the internal and external balance of the human body (Brody, 2020; O’Riordan et al., 2025). With the rapid advancements in sequencing technology, there has been increasing recognition of the gut microbiota’s critical role in human health, including its impact on male sexual function. Studies have established a link between intestinal endotoxins and a decline in gonadal function, affecting testosterone secretion and sperm quality, which can result in reduced fertility and quality of life (Tremellen, 2016). Conversely, probiotics have been shown to enhance the intestinal barrier, reduce metabolic endotoxemia and inflammation, and subsequently improve testosterone levels (Guo et al., 2024). Furthermore, a significant body of evidence highlights the close association between gut microbiota and the development of various metabolic diseases, including diabetes, hypertension, hyperlipidemia, and obesity (Crudele et al., 2023; Li and Chen, 2023; Jia et al., 2023). These metabolic disorders are recognized as major risk factors for the development of erectile dysfunction (Wang et al., 2025), suggesting a potential link between gut microbiota and the onset of ED. In addition, an imbalance in the gut microbiota can lead to the proliferation of potentially pathogenic bacteria, disrupting immune homeostasis and triggering pro-inflammatory responses. Metabolites of gut microbiota, such as lipopolysaccharides from the cell walls of Gram-negative bacteria, are considered endotoxins that cause inflammation (Di Vincenzo et al., 2024). In dysbiosis, lipopolysaccharides released from the death and lysis of intestinal bacteria bind to Toll-like receptor 4 (TLR4) on intestinal epithelial cells, activating the release of inflammatory cytokines (e.g., interleukins, tumor necrosis factor). The abnormal accumulation of these inflammatory factors leads to a chronic inflammatory state, causing endothelial dysfunction and potentially contributing to ED (Li et al., 2023; Ji et al., 2024). However, only a limited number of studies have utilized 16S rRNA gene sequencing to explore the microbial composition in ED patients (Okamoto et al., 2020; Geng et al., 2021; Kang et al., 2024), and there is still a lack of comprehensive understanding of species-level information in the gut microbiota and the associated metabolic pathways.

In light of the above, the present study utilized macro-genomic sequencing to investigate the changes in gut microbiota and metabolic pathways between Chinese ED patients and healthy individuals. The aim was to compare the changes in gut microbiota and metabolic pathways between ED males and healthy control group.

## 2 Matirials and methods

### 2.1 Study design

This was a case-control study. A total of 19 male patients diagnosed with ED were recruited from the Department of Reproductive Men’s Medicine at the First Affiliated Hospital of Dalian Medical University between May and July 2023. The diagnostic criteria for ED were based on the 5-item version of the International Index of Erectile Function (IIEF-5). Additionally, 15 healthy men with normal sexual function were recruited as controls. Inclusion criteria for participants were as follows: (1) aged between 18 and 60 years; (2) heterosexual, with a regular sexual partner and a stable sexual life for at least 3 months; and (3) normal external genitalia development with no history of trauma. Participants were excluded if they met any of the following criteria: (1) use of antimicrobials, probiotics, or any gastrointestinal disease treatment in the past month; (2) history of diabetes mellitus, hypertension, or thyroid dysfunction; (3) previous radical prostatectomy, pelvic trauma, or surgery; or (4) a history of severe psychiatric disorders such as anxiety, depression, or other serious mental illnesses.

The study was reviewed and approved by the Ethics Committee of the First Affiliated Hospital of Dalian Medical University (PJ-KS-KY-2023-167), and all participants provided written informed consent.

### 2.2 Clinical information collection and analysis

Demographic and clinical information was collected through questionnaires and electronic medical records. The data collected included age, body mass index (BMI), International Prostate Symptom Score (IPSS), testosterone, estradiol, follicle-stimulating hormone (FSH), luteinizing hormone (LH), prolactin, glucose, HDL, LDL, triglycerides, total cholesterol, homocysteine, and lipoproteins.

### 2.3 Fecal specimen collection and pretreatment

Fecal samples were collected from all participants using sterile plastic spoons and stored in sterile plastic tubes. The samples were promptly placed in a –20°C refrigerator and subsequently transferred to a –80°C freezer within 24 h for long-term storage.

### 2.4 Gene sequencing and data analysis

(1) Genomic DNA was extracted from fecal samples using the Tengen Magnetic Bead Kit. (2) DNA purity and integrity were assessed via 1% agarose gel electrophoresis, and DNA concentration was quantified using the Qubit^®^ dsDNA Assay Kit in the Qubit^®^ 2.0 Fluorometer (Life Technologies, CA, United States). The appropriate amount of sample was placed in a centrifuge tube, diluted with sterile water, and adjusted to an OD value between 1.8 and 2.0. (3) Library construction involved using 1 μg of genomic DNA, which was randomly fragmented into ∼350 bp pieces using a Covaris Ultrasonic Crusher. Following end repair, A-tail addition, sequencing junction addition, purification, PCR amplification, and other steps, the library was completed. After library construction, preliminary quantification was performed using Qubit 2.0, and the library was diluted to 2 ng/μL. The insert size was assessed using the Agilent 2100 system. After confirming the insert size met expectations, Q-PCR was employed to accurately determine the effective concentration of the library (with a required concentration > 3 nM) to ensure high-quality output. Once the library passed quality control, different libraries were pooled according to their effective concentration and target sequencing volume for PE150 sequencing.

### 2.5 Bioinformatics analysis

(1) The raw data obtained from the NovaSeq sequencing platform were preprocessed using fastp to generate clean data for subsequent analysis. The clean data were then assembled and analyzed using MEGAHIT software (Karlsson et al., 2012); (2) MetaGeneMark was employed to predict the open reading frames (ORFs) for each sample, and the results were de-redundant using CD-HIT software to generate a non-redundant initial gene catalog (Mende et al., 2012). Bowtie2 was used to align the clean data from each sample to the initial gene catalog, and the final gene catalog (non-redundant) was obtained after filtering. The final gene catalog (unigenes) was used for further analysis (Qin et al., 2010); (3) The unigenes were annotated against Micro_NR sequences and the KEGG database using DIAMOND software (Buchfink et al., 2015). Alpha diversity was assessed using Shannon and Simpson indices. Principal component analysis (PCA) was performed, and differences between groups were examined using ANOSIM analysis. MetaGenomeSeq and LEfSe analyses were then conducted to identify species that differed between groups (Feng et al., 2015). MetaGenomeSeq analysis was used to perform hypothesis testing between groups for each taxonomic stratum to obtain p-values and Q-values, while LEfSe analysis was performed using LEfSe software (LDA score set to 2 by default) (Segata et al., 2011). Random forest algorithms were applied for regression analyses (R-pROC and randomforest packages) (Deo, 2015), and random forest models were constructed by selecting species by gradient at the species level. Important species were screened by MeanDecreaseAccurance, and then each model was cross-validated (default 10-fold) to plot ROC curves.

### 2.6 Statistical analysis

Statistical analysis was performed using SPSS26 and version 3.6.1 R software. Comparisons between the two groups were made using the independent samples *t*-test and were expressed as ($\bar{\text{x}}$ ± s). *p* < 0.05 was considered statistically significant.

## 3 Results

### 3.1 Clinical characteristics of the participants

A total of 19 ED patients and 15 healthy controls participated in this study. As shown in Table 1, there were no significant differences between the two groups in terms of age and body mass index. Regarding sex hormone levels, no significant differences were observed between the groups in testosterone, estrogen, or prolactin levels. Similarly, for biochemical indices, no significant differences were found in the levels of total cholesterol (TC) and triglycerides (TG) between the two groups.

**TABLE 1:** The demographics and serum characteristics of patients with ED and healthy controls.

Cohort characteristic	ED group	Control group	*t*	*P*-value
Age (y)	31.16 ± 3.11	29.73 ± 5.12	0.948	0.35
BMI (kg/m^2^)	27.37 ± 7.21	23.47 ± 3.25	1.944	0.06
IIEF-5	12.11 ± 4.9	22.53 ± 2	–8.436	<0.001
IPSS	4.21 ± 5.26	2.4 ± 4.87	1.030	0.31
Testosterone (ng/mL)	5.33 ± 2.6	5.85 ± 1.88	–0.645	0.52
Estradiol (pg/mL)	27.67 ± 7.35	24.85 ± 3.82	1.443	0.160
Follicle stimulating hormone (mIU/mL)	5.53 ± 2.32	4.53 ± 1.63	1.417	0.17
Luteinizing hormone (mIU/mL)	4.81 ± 1.86	4.62 ± 1.33	0.323	0.75
Prolactin (μIU/mL)	219.5 ± 95.1	279.59 ± 137.65	–1.504	0.14
Blood sugar (mmol/L)	7.41 ± 4.16	5.37 ± 0.65	2.109	0.05
HDL (mmol/L)	1.27 ± 0.26	1.25 ± 0.2	0.188	0.85
LDL (mmol/L)	2.79 ± 0.51	2.67 ± 0.82	0.513	0.61
Triglyceride (mmol/L)	2.25 ± 2.33	1.68 ± 0.97	0.879	0.39
Total cholesterol (mmol/L)	5.06 ± 0.73	4.8 ± 1.06	0.852	0.40
Homocysteine (mmol/L)	17.86 ± 10.73	23.62 ± 22.41	–0.916	0.37
Lipoprotein (mmol/L)	162.21 ± 79.37	156.52 ± 150.79	0.132	0.90

### 3.2 Alterations in the diversity and structure of gut microbiota in ED patients

**FIGURE 1:**
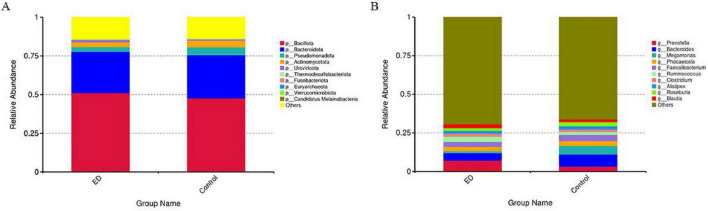
Comparisons of the gut microbial community compositions. **(A)** Relative proportions of bacterial phyla in ED and control groups. Bacillota, Bacteroidota, Pseudomonadota, Actinomycetota, Uroviricota, Thermodesulfobacteriota, Fusobacteriota. Euryarchaeota, Verrucomicrobiota, and Candidatus Melainabacteria are presented in different colors, other phyla are categorized as “Other.” **(B)** Relative proportions of bacterial genus in ED and control groups. Prevotella, Bacteroides, Megamonas, Phocaeicola, Faecalibacterium, Ruminococcus, Clostridium, Alistipes, Roseburia, and Blauti Presented in different colors, other genus are categorized as “Other”.

We investigated changes in the composition of the gut microbiota at both the “phylum” and “genus” levels between ED patients and controls. At the phylum level, the two most abundant microbiota in the gut of both groups were Bacillota (51.1% vs. 47.6%) and Bacteroidota (26.6% vs. 28.0%). At the genus level, Prevotella (9.8%) and Bacteroides (5.1%) were the two most abundant genera in ED patients, while in the control group, Bacteroides (7.8%) had the highest abundance, followed by Prevotella (3.3%) (Figures 1a,b).

**FIGURE 2:**
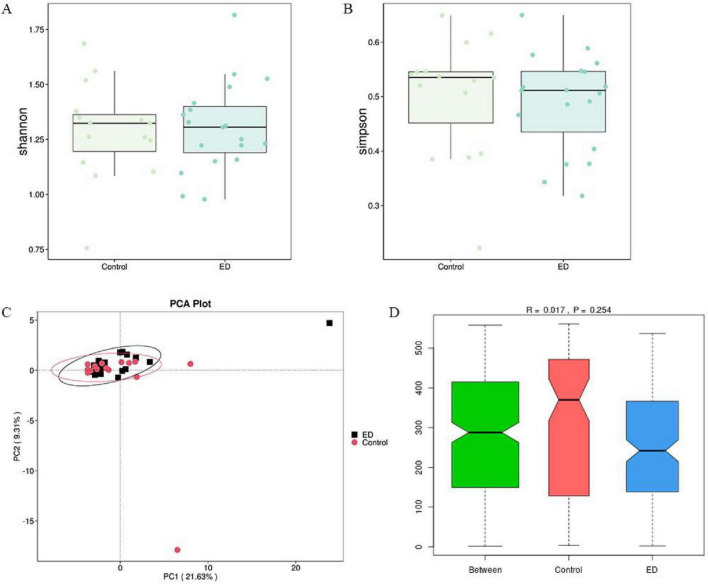
Comparison of microbial diversity. **(A,B)** There was no significant difference in the diversity of gut microbiota (Shannon and Simpson) between the ED and control groups. **(C,D)** PCA analysis based on unweighted UniFrac distances showed no significant difference in overall microbial diversity between the ED and control groups (ANOSIM, *R* = 0.017, *p* = 0.26).

To compare the microbial community richness and homogeneity between the two groups, we analyzed the samples for alpha diversity. The results showed no significant differences in gut microbiota diversity (Shannon and Simpson indices) between the ED and control groups (*p* = 0.8 vs. *p* = 0.4). However, there was a trend toward decreasing alpha diversity in the ED patients (Figures 2A,B). Furthermore, we performed β-diversity analysis to assess the differences in microbial community composition and distribution between the groups. The results revealed no significant separation between the two groups after clustering the ED group (black) and the control group (red). The unweighted UniFrac distance also showed no statistically significant difference (*R* = 0.017, *p* = 0.26), indicating that there were no significant structural changes in the gut microbiota between the two groups (Figures 2C,D).

### 3.3 Identification of key gut microbiota between ED and control groups

**FIGURE 3:**
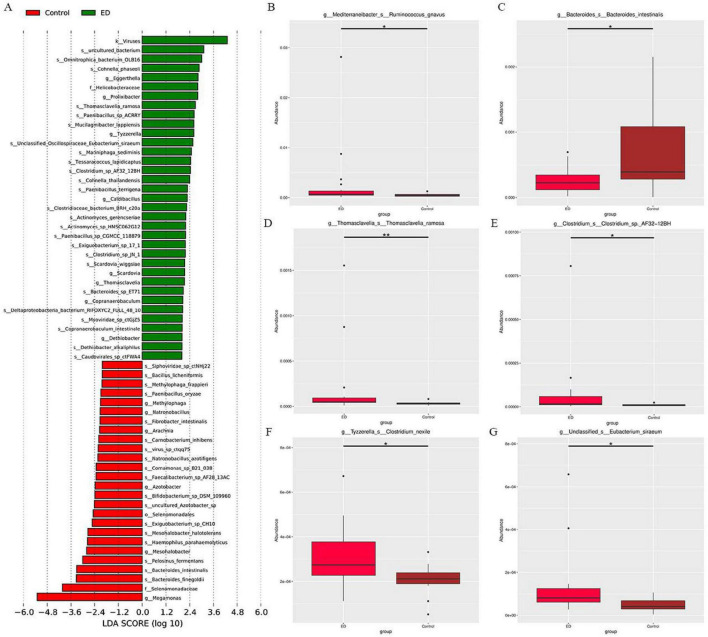
**(A)** LEfSe analysis identified gut microbiota with significant differences in abundance between the ED and control groups (LDA score > 2, p < 0.05). **(B-G)** Metastate analysis showed that Ruminococcus gnavus, Thomasclavelia ramosa, Clostridium sp. AF32-12BH, Clostridium nexile, Eubacterium siraeum and Bacteroides intestinalis abundance differed significantly between ED and control groups (*p < 0.05, **p < 0.01).

To identify the species that most significantly contributed to the differences between the two groups, LefSe analysis was conducted on specimens from both the ED and control groups. A total of 61 gut microorganisms were found to be significantly different, with 35 species being more abundant in the ED group and 26 in the control group (Figure 3A). Furthermore, a comparison of the abundance of the main species revealed that in the ED group, Ruminococcus gnavus, Thomasclavelia ramosa, *Clostridium* sp. AF32-12BH, Clostridium nexile, and Eubacterium siraeum were more abundant, while the abundance of Bacteroides intestinalis was significantly reduced compared to the control group (Figures 3B–G).

### 3.4 Differential analysis of gut microbial metabolic pathways

Metabolic pathways of the gut microbiota were predicted, and those with differential expression between the controls and ED patients were identified through LEfSe analysis. As shown in Table 2, pathways associated with nucleotide and lipid metabolism, such as purine metabolism (ko00230) and glycerophospholipid metabolism (ko00564), were more highly expressed in the gut microbiota of ED patients. In contrast, pathways related to amino acid and vitamin metabolism, such as valine, leucine, and isoleucine degradation (ko00280) and lipoic acid metabolism (ko00785), were more abundant in the control group (*P* < 0.05).

**TABLE 2:** Linear discriminant analysis effect size (lefse) analysis of two groups of pathway abundance.

Characteristic	ID	Name	Class	LDA	*P*-value
ED	ko00230	Purine metabolism	Metabolism; nucleotide metabolism	2.697	0.006
	ko00564	Glycerophospholipid metabolism	Metabolism; lipid metabolism	2.354	0.030
	ko00983	Drug metabolism - other enzymes	Metabolism; xenobiotics biodegradation and metabolism	2.339	0.042
	ko00770	Pantothenate and CoA biosynthesis	Metabolism; metabolism of cofactors and vitamins	2.222	0.036
	ko05012	Parkinson disease	Human diseases; neurodegenerative disease	2.173	0.033
	ko05418	Fluid shear stress and atherosclerosis	Human diseases; cardiovascular disease	2.157	0.033
Control	ko00280	Valine, leucine and isoleucine degradation	Metabolism; amino acid metabolism	2.365	0.004
	ko00785	Lipoic acid metabolism	Metabolism; metabolism of cofactors and vitamins	2.064	0.023
	ko05230	Central carbon metabolism in cancer	Human diseases; cancer: overview	2.156	0.021

### 3.5 Predictive modeling of gut microbes

**FIGURE 4:**
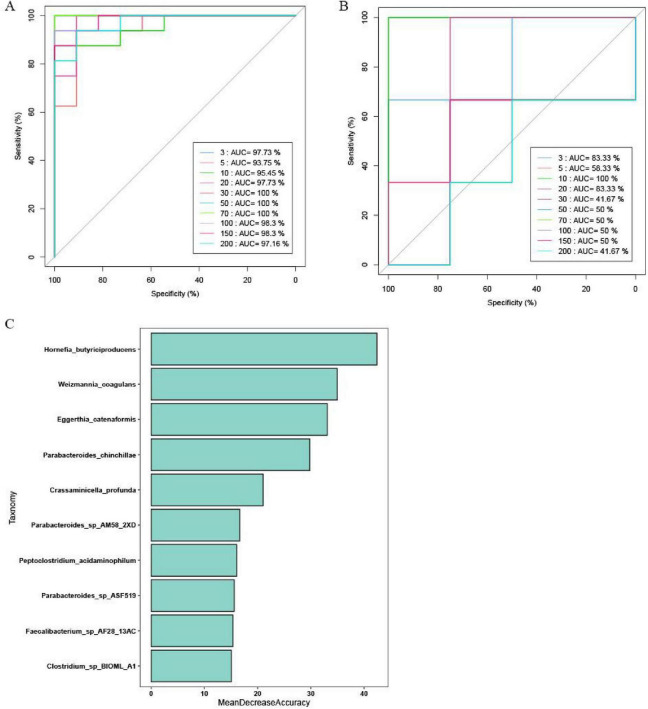
The gut microbiota classifier for ED. The ROC curve reveals the AUC values of prediction models with different species in **(A)** Training set and **(B)** Validation set. **(C)** The bacterial genera that could significantly discriminate between the control group and the ED group were presented in descending order.

To explore the potential of gut microbes as biomarkers for ED, we developed a diagnostic model using the random forest algorithm. The model, based on 10 species, demonstrated high performance, with AUC values of 95.45 and 100% for the training and validation sets, respectively (Figures 4A,B). These results indicate that the model can accurately differentiate between ED patients and healthy controls. Additionally, the species identified as significant by the model were highlighted using mean decreasing accuracy (Figure 4C).

## 4 Discussion

The human gut microbiota consists primarily of six major groups: thick-walled bacilli, anaplasma, actinobacteria, Mycobacterium, and Clostridium, with the dominant groups being anaplasma and thick-walled bacilli (Trakman et al., 2022). These microbiota play a key role in regulating the body’s immunity, metabolism, and endocrine processes, primarily through their own activities and metabolic products (Morrison and Preston, 2016).

We observed differences in the abundance of certain gut microbiota between the control and ED groups through macro gene sequencing. Specifically, the abundance of Ruminococcus gnavus, Thomasclavelia ramosa, *Clostridium* sp. AF32-12BH, Clostridium nexile, and Eubacterium siraeum species was increased in ED patients, while the abundance of Bacteroides intestinalis decreased. Previous studies have linked Thomasclavelia ramosa, a species associated with ecological disorders, to non-alcoholic fatty liver disease (Mbaye et al., 2023). Clostridium ramosa, a Gram-positive bacterium belonging to the phylum Firmicutes that forms spores and grows in anaerobic environments (She et al., 2024), has been found to be closely associated with fructose and bile acid metabolism, suggesting it may be a potential target for treating non-alcoholic steatohepatitis (Zhang et al., 2023). Zhu et al. conducted a Mendelian randomization analysis exploring the causal relationship between gut microbiota and ED, finding that Tyzzerella3 species were linked to an increased risk of ED (Zhu et al., 2024). In a study by Smadar Shilo et al., LefSe analysis revealed that Eubacterium siraeum was significantly enriched in individuals with type 1 diabetes (Shilo et al., 2022). Hu et al. compared the gut microbiome at the species level and found that Ruminococcus gnavus was enriched in the obese group (Hu et al., 2024). Summarizing, the gut microbes identified in our study, which showed increased abundance in ED patients, appear to influence glucose and lipid metabolism, but their precise biological roles in ED development remain to be further explored.

Notably, combining the results from LEfSe and Metastat analyses, only the abundance of Bacteroides intestinalis was significantly reduced in the ED group (Bakir et al., 2006). Previous studies have shown that Enterobacter aerogenes degrades complex arabinoxylans and xylans found in dietary fibers such as wheat, rye, oats, and barley (Dhingra et al., 2012). The degradation products, butyrate and ferulic acid, have demonstrated various functional activities, including antioxidant, anti-inflammatory, and antimicrobial effects (Cortés-Martín et al., 2020; Shen et al., 2022). These metabolites can interact with the immune system, modulating the development and function of almost all types of immune cells in the intestinal immune cell pool, thereby influencing immune function and preventing overactive immune responses (Golpour et al., 2023; Zhang et al., 2022). Therefore, we speculate that the reduced abundance of Bacteroides intestinalis may contribute to a prolonged inflammatory state in the body, impairing the vascular endothelium and promoting the development of ED. However, the exact mechanism remains to be confirmed. Additionally, previous studies have found that supplementing with polysaccharides from Lyophyllum mushrooms can reduce obesity and hyperlipidemia in mice by increasing the abundance of Bacteroides intestinalis (Wang et al., 2022). Thus, Bacteroides intestinalis may represent a potential therapeutic target for ED in the future.

There is some controversy regarding changes in the diversity of the gut microbiota in ED patients. Qiao et al.’s study found higher alpha diversity (Shannon index) in the ED group (Qiao et al., 2024), which contrasts with Geng et al.’s findings (Geng et al., 2021). In contrast, Kang et al.’s study presented a third conclusion, reporting no significant difference between the ED and control groups across several indices, including species richness (Chao1 and observed OTUs) and diversity of gut microbiota (Shannon and Simpson indices) (Kang et al., 2024). In the present study, the ED group showed a trend toward lower alpha diversity, but this difference was not statistically significant. Notably, unlike previous studies, no statistically significant difference in β-diversity was observed between the two groups, suggesting that there may be no significant difference in the microbiota composition between the groups. We have conducted a comprehensive analysis to explore the reasons for the differences in research results. Firstly, a small sample size may be a key factor contributing to the discrepancies. Secondly, the different stages of ED could also impact the characteristics of the gut microbiota. In the early stages of the disease, the abundance of gut microbiota decreases, leading to a significant reduction in diversity. However, as the disease progresses, the diversity of the gut microbiota may stabilize through compensatory regulation, changes in dietary structure, and drug treatments. Additionally, differences in dietary habits among populations may contribute to variability in findings (Bibbò et al., 2016). Newman et al. noted that diet is a critical factor influencing gut bacterial diversity (Newman et al., 2021). The diversity results observed in our study are similar to those of Kang et al. (2024), which may be due to the fact that our study sample is from northern China, where dietary patterns are similar. Given these factors, particularly the controversial results regarding microbiota diversity in ED patients, it is essential to expand the sample size in future studies and minimize the impact of regional differences. This approach will help yield more reliable and comprehensive conclusions.

Our study compared the functional genes of the gut microbiota using the KEGG database, and the results indicated that the functions of microbiota with significant changes in ED were primarily reflected in the upregulation of genes related to atherosclerosis, purine metabolism, and glycerophospholipid metabolism, along with the downregulation of genes involved in lipoic acid metabolism, and valine, leucine, and isoleucine degradation. Branched-chain amino acids (BCAAs) leucine, isoleucine, and valine are essential amino acids that cannot be produced by the human body and must be obtained through the diet (McGarrah and White, 2023). These amino acids play a crucial role in various metabolic processes, including energy generation and utilization, protein synthesis and breakdown, fat conversion, and sugar metabolism. As early as 1960, studies noted an increase in BCAA levels in obese patients, and with the advent of metabolomics techniques, the correlation between obesity, insulin resistance, and BCAAs has been further confirmed (Newgard et al., 2009; Felig et al., 1969). Vanessa et al. found that transplanting fecal microbiota from obese twins into germ-free mice resulted in higher circulating BCAA levels in the mice compared to those receiving microbiota from lean twins (Ridaura et al., 2013). This suggests that a reduction in gut microbiota capable of biodegrading BCAAs could lead to elevated circulating BCAA levels, potentially contributing to obesity and glucose metabolism disorders. Additionally, the ED group showed significant upregulation of functional genes related to lipid metabolism, particularly glycerophospholipid metabolism. This finding is consistent with previous literature, which reports that diabetes, hypertension, dyslipidemia, and obesity are independent risk factors for ED (Yafi et al., 2016).

There are some limitations in this study that should be addressed in future research. First, as a pilot study, the sample size was small, and additional cases are needed to confirm our findings. Second, ED can be categorized into cardiac, organic, and mixed types based on underlying pathogenesis. However, the ED patients in this study were not specifically categorized, and thus, we could not compare microbiota changes between these subgroups. Finally, as mentioned earlier, the study sample has certain geographical characteristics. Although we established strict inclusion criteria, it is difficult to fully exclude the impact of dietary factors or other drugs on the gut microbiota.

## 5 Conclusion

This study found no significant difference in the diversity and structure of the gut microbiota between the ED population and healthy individuals. However, changes were observed in the abundance of certain gut microbiota, notably a significant decrease in Bacteroides intestinalis and an increase in Ruminococcus gnavus in the ED group. These microbial alterations may contribute to the increased risk of ED by promoting atherosclerosis, enhancing lipid metabolism, and inhibiting the degradation of branched-chain amino acids. Improving the intestinal microecology could potentially become a novel approach for the prevention and treatment of ED in the future.

## Data Availability

The original contributions presented in the study are publicly available. This data can be at: https://www.ncbi.nlm.nih.gov/sra/PRJNA1261102.
